# NeurodegenERation: The Central Role for ER Contacts in Neuronal Function and Axonopathy, Lessons From Hereditary Spastic Paraplegias and Related Diseases

**DOI:** 10.3389/fnins.2019.01051

**Published:** 2019-10-11

**Authors:** Philippa C. Fowler, M. Elena Garcia-Pardo, Jeremy C. Simpson, Niamh C. O’Sullivan

**Affiliations:** ^1^UCD School of Biomolecular and Biomedical Science, UCD Conway Institute, University College Dublin, Dublin, Ireland; ^2^UCD School of Biology and Environmental Science, UCD Conway Institute, University College Dublin, Dublin, Ireland

**Keywords:** endoplasmic reticulum, axon, mitochondria, lipid droplet, endolysosome, plasma membrane, microtubule

## Abstract

The hereditary spastic paraplegias (HSPs) are a group of inherited neurodegenerative conditions whose characteristic feature is degeneration of the longest axons within the corticospinal tract which leads to progressive spasticity and weakness of the lower limbs. Though highly genetically heterogeneous, the majority of HSP cases are caused by mutations in genes encoding proteins that are responsible for generating and organizing the tubular endoplasmic reticulum (ER). Despite this, the role of the ER within neurons, particularly the long axons affected in HSP, is not well understood. Throughout axons, ER tubules make extensive contacts with other organelles, the cytoskeleton and the plasma membrane. At these ER contacts, protein complexes work in concert to perform specialized functions including organelle shaping, calcium homeostasis and lipid biogenesis, all of which are vital for neuronal survival and may be disrupted by HSP-causing mutations. In this article we summarize the proteins which mediate ER contacts, review the functions these contacts are known to carry out within neurons, and discuss the potential contribution of disruption of ER contacts to axonopathy in HSP.

## Introduction

Hereditary spastic paraplegias (HSPs) are a genetically complex group of neurodegenerative disorders characterized by degeneration of the longest axons within the corticospinal tract leading to progressive lower limb spasticity and weakness. To date, mutations in over 80 spastic paraplegia genes (SPGs) and 60 gene products have been identified to give rise to HSP (Blackstone, 2018). Proteins encoded by these SPGs have roles in an apparently diverse range of cellular functions including: organelle shaping, axonal transport, lipid metabolism, mitochondrial function and endosomal trafficking. Despite this genetic and functional diversity, it has become clear over the last decade that the most prevalent SPGs, accounting for over half of all cases of autosomal dominant HSP, encode proteins that are involved in the shaping and organization of the tubular endoplasmic reticulum (ER) (Boutry et al., 2019).

**FIGURE 1:**
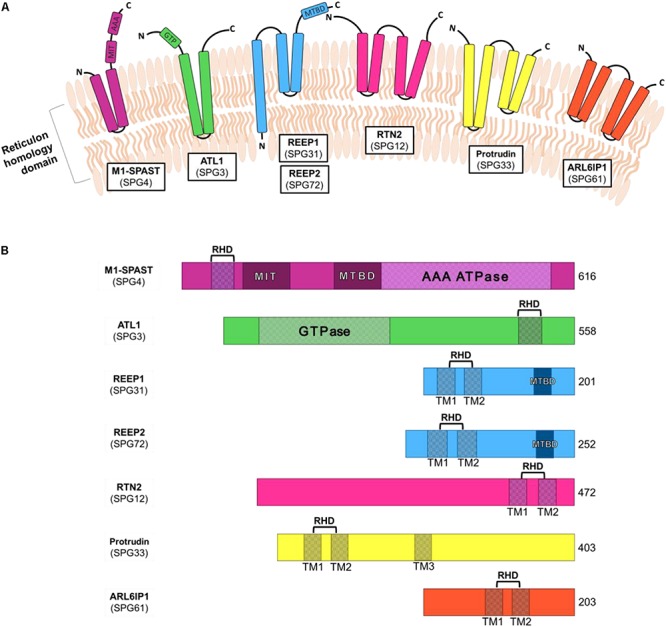
Schematic diagrams of the structural domains of HSP-causing ER-associated genes. **(A)** ER-shaping proteins insert into the ER membrane via the reticulon homology domain. **(B)** ER-shaping protein domains include reticulon homology domain (RHD), made up of one or more transmembrane regions (TM), as well as microtubule interacting domain (MIT), microtubule binding domain (MTBD) and ATPase associated with various cellular activities (AAA). Shown are the amino acid number for the full-length longest isoforms of each protein.

The ER-shaping proteins are integral membrane proteins which localize to the outer layer of the ER bilayer and regulate the organization of the tubular ER network. These proteins generally share little sequence homology, however, they all possess a reticulon homology domain (RHD) (Figure 1). This conserved domain consists of two hydrophobic sequences separated by a hydrophilic linker that forms a wedge by which the proteins insert into the lipid bilayer (Voeltz et al., 2006; Hu et al., 2008). By forming homomeric and heteromeric oligomers the ER-shaping proteins shape and stabilize the highly curved ER tubules (Shibata et al., 2009). Many different ER-shaping proteins are known including: Spastin (Park et al., 2010), Atlastin (Hu et al., 2009), REEP (Shibata et al., 2008) and Reticulon (Voeltz et al., 2006). Mutations in any one of these genes causes axonopathy and gives rise to HSP (Hazan et al., 1999; Zhao et al., 2001; Züchner et al., 2006b; Montenegro et al., 2012; Esteves et al., 2014). It is therefore evident that the tubular ER has a vital role in the maintenance of long axons. However, the axonal functions of tubular ER are not well understood and mechanism(s) by which disruption of this organelle by HSP-causing mutations leads to neurodegeneration is not known.

**FIGURE 2:**
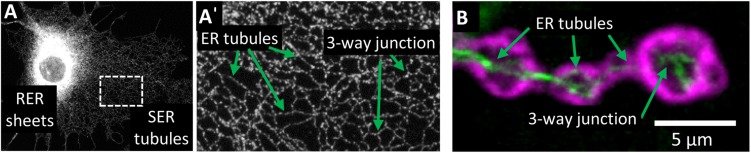
Organization of the tubular ER network. **(A)** Visualization of ER compartments in Cos7 cells stained with REEP5 antibody. Rough ER (RER) sheets radiate from the nuclear envelope and are densely packed in the perinuclear region. Smooth ER (SER) tubules form a highly interconnecting network that extends to the periphery of the cell **(A’)**. **(B)** Visualization of ER tubules within terminal boutons of *Drosophila melanogaster* motor neurons expressing Rtnl1:YFP (green) and stained with post-synaptic density protein DLG antibody (magenta). Within neurons, ER tubules extend the length of the axon and into the terminal boutons.

The ER network is morphologically and functionally compartmentalized within different areas of cells, none more so than in highly polarized neurons (Figure 2). Within the neuronal cell body, ribosome-studded rough-ER (RER) predominates, the primary function of which is likely protein synthesis (Broadwell and Cataldo, 1983; Rolls, 2002; Wu et al., 2017). ER-shaping proteins are localized only at the curved edges of RER nanoholes and sheets (Shibata et al., 2010; Schroeder et al., 2019). In contrast to the cell body, ribosome-studded RER is not observed within the long axonal projections of neurons. Instead, tubules of smooth ER (SER) can be seen to extend the entire length of the axon into the presynaptic boutons (Wu et al., 2017; Yalçın et al., 2017). Throughout the axon these ER tubules make extensive contacts with other organelles and the cytoskeleton. At these contact sites, organelle membranes remain closely tethered but do not fuse. ER contacts are highly dynamic yet are not merely fleeting glances and can be maintained during organelle movement or trafficking (Friedman et al., 2010). Through these interactions, the tubular ER can regulate an array of cellular functions. Moreover, mutations in the genes encoding the protein tethers that mediate these contacts cause axonopathy in HSP and other neurodegenerative diseases including amyotrophic lateral sclerosis (ALS) and Charcot-Marie-Tooth disease (CMT) (Table 1). It is therefore important that we further our understanding of ER contacts to understand the role of tubular ER in axons and axonopathy.

**TABLE 1:** Overview of the proteins mediating tubular ER contacts and their involvement in neurodegenerative disease.

**Protein**	**Subcellular localization**	**Function at ER contact**	**Causative link to neurological disease (if known)**	**References**
**ER-mitochondrial contacts**				
VAPB	ER	VAPB and PTPIP51 interact forming tethers between the ER and mitochondria	Mutations in VAPB cause ALS type-8	Nishimura et al., 2004
PTPIP51	Mitochondria			
MFN2	ER, mitochondria	MFN2 contributes to the maintenance of ER-mitochondrial contacts through homo- or hetero-meric interactions with MFN1	Mutations in MFN2 cause CMT type 2A and hereditary motor and sensory neuropathy type 2A (HMSN2A)	Züchner et al., 2004; Züchner et al., 2006a
MFN1	Mitochondria			
PDZD8	ER	PDZD8 mediates ER-mitochondrial contacts by a mechanism that is yet to be determined and regulates Ca^2+^ transfer		
**ER-lipid droplet (LD) contacts**				
Seipin	ER/LD	Seipin dodecamers mediate ER-LD contacts and function to transfer lipids from the ER into bound LDs	Mutations in Seipin cause SPG17	Windpassinger et al., 2004
Snxl4	ER/LD	Snxl4 forms a tether between ER and LDs	Mutations in Snxl4 cause	Thomas et al., 2014
			spinocerebellar ataxia autosomal recessive 20	
Rabl8	ER/LD	Rabl8-DFCP1 complexes form tethers between ER and LDs	Mutations in Rabl8 cause Warburg Micro syndrome	Bem et al., 2011
DFCP1	ER/LD			
**ER-endolysosome contacts**				
VAPA/B Mospd2	ER	Interactions between ER proteins VAPA/B or MOSPD2 and FFAT domain proteins (ORP1L, StARD3, StARD3NL) mediate	Mutations in VAPB cause ALS type-8	Nishimura et al., 2004
F FAT domain proteins	Late endosome	ER-eondosomal contacts which contribute to endosomal fission, endosomal trafficking and sterol transfer		
Protrudin	ER	Protrudin interacts with Rab7 and PI3P to promote ER- endome contacts which regulates endosomal trafficking along neurites	A mutation in Protrudin is associated with SPG33, however, the pathogenicity of the mutation is debated; Mutations in Rab7 cause CMT type 2B	Verhoeven et al., 2003; Mannan et al., 2006; Martignoni et al., 2008
Rab7	Endosome			
PTP1B	ER	PTP1B promotes ER-endosomal contacts by interacting with endosomal proteins such as EGFR		
EGFR	Endosome			
**ER-plasma membrane (PM) contacts**				
E-Sytl-3	ER	E-Syts mediate ER-PM contact and may function to regulate Ca^2+^ and lipid transfer		
VAPA/B	ER	VAPA/VAPB interactions with Kv2.1/2.2 form ER-PM contacts which act as traffickin hubs for intracellular signaling	Mutations in VAPB cause ALS type-8	Nishimura et al., 2004
Kv2.1/2.2	PM			
TMEM24	ER	TMEM24 mediates ER-PM contacts by electrostatic interaction and may regulate lipid transfer		
**ER-microtubule (MT) contacts**				
CLIMP-63	ER	CLIMP-63 binds MTs forming ER-MT contacts which function to regulate ER distribution in axons		
pl80	ER	pl80 binds MTs forming ER-MT contacts which function to stabilize MTs		
Sec61β	ER	Sec61β binds MTs forming ER-MT contacts which function to organize ER tubules along MTs		
REEP1	ER	REEP1 binds MTs forming ER-MT contacts which function to organize ER tubules along MTs	Mutations in REEP1 cause SPG31	Züchner et al., 2006b
Ml-Spastin	ER	Ml-Spastin binds and severs MTs regulating MT organization and regrowth	Mutations in Spastin cause SPG4	Hazan et al., 1999
STIM1/2	ER	STIM1/2-EB1 interactions for ER-MT contacts which regulate the transport of ER tubules along growing MTs		
EB1	MT			

The aim of this article is to review the current studies examining the functions of tubular ER in neurons and to highlight the areas where more work is urgently needed to further our understanding of the mechanism(s) by which disruption of this organelle may cause pathogenicity in HSP. We focus on discussing ER contacts with organelles and cytoskeletal elements for which there is the strongest evidence of a role in neurons.

## ER-Mitochondrial Contacts in Neurons

Contact sites between the ER and mitochondria have been observed in electron micrographs since the 1950s, with those early experiments revealing a number of electron dense structures that localize to specialized domains between the ER and mitochondria (Copeland, 1959). These mitochondria-associated ER membranes (MAMs) are maintained at ∼10–30 nm, depending on cell type and the level of stress to which the cells are exposed, and it is estimated that ∼5–12% of the outer mitochondrial surface remains connected to the ER (Csordás et al., 2006; Stoica et al., 2014; Wu et al., 2017). This tight association suggests that maintenance of ER-mitochondrial contacts are vital for the function of both organelles and the cell more generally, and many studies have focused on elucidating the molecular complexes that are involved in ER-mitochondrial tethering.

### How Are ER-Mitochondrial Contacts Regulated?

**FIGURE 3:**
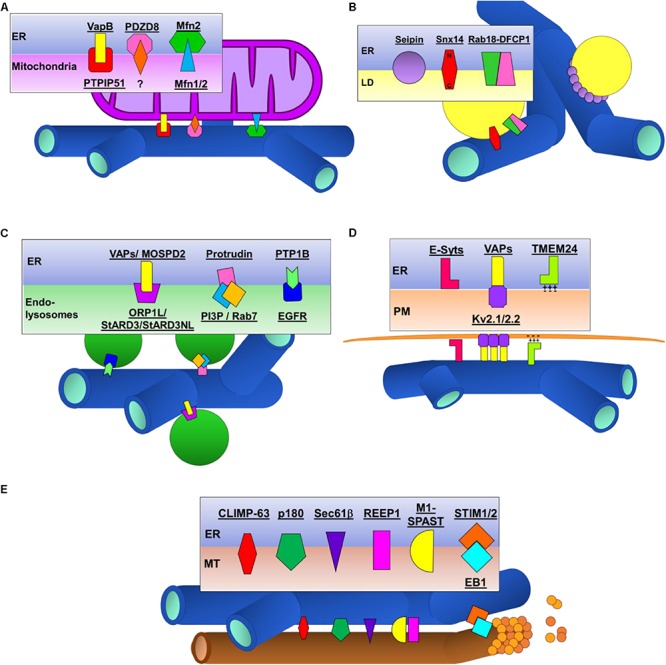
Schematic illustrations of proteins tethers that contact the ER to other organelles and the cytoskeleton. **(A)** ER-mitochondrial contacts. **(B)** ER-lipid droplet (LD) contacts. **(C)** ER-endolysosomal contacts. **(D)** ER-plasma membrane (PM) contacts. **(E)** ER-microtubule (MT) contacts.

In yeast, the ER-mitochondria encounter surface (ERMES) and the ER membrane protein complex (EMC) proteins that mediate tethering of ER and mitochondria are well established (Kornmann et al., 2009; Lahiri et al., 2014). The identity of ER-mitochondrial tethers in eukaryotic cells however, has been much more complicated. In part, this is due to the large number of proteins that localize to ER-mitochondrial contacts (transporters, enzymes, receptors etc.) most of which are not in fact tethers. Distinguishing between these and *bona fide* tethers required to mediate the contact of these two organelles has proved challenging. Here we will discuss the most recent findings on the best studied of the proposed ER-mitochondrial tethers Mfn2, VAPB-PTPIP5 and PDZD8 (Figure 3A).

Mitofusins 1 and 2 (Mfn1 and Mfn2) are GTPases which regulate fusion of the outer mitochondrial membrane (OMM) during mitochondrial fusion. More recently, it has been shown that ER-resident Mfn2 also plays an important role in regulating ER-mitochondrial tethering. The first study identifying this novel role of Mfn2 outside the mitochondria used confocal microscopy to detect a disruption in ER morphology and a decrease in the distance between the ER and mitochondria in Mfn2 knockout cells (De Brito and Scorrano, 2008). The same group has more recently used electron microscopy (EM) and fluorescence-based proximity probes to further support their assertion that Mfn2 acts as an ER-mitochondrial tether via homodimers or heterodimers with Mfn1 (Naon et al., 2016). However, the exact nature of Mfn2’s role in ER-mitochondrial contacts remains debated as several independent studies have found that loss of Mfn2 results in an increase in ER-mitochondrial association (Cosson et al., 2012; Filadi et al., 2015; Leal et al., 2016). These conflicting findings have not yet been fully explained, however, it is worth considering that under different conditions and with localized variations in protein concentrations, Mfn2 may contribute differently to the maintenance of ER-mitochondrial contacts.

Probably the best studied ER-mitochondrial tether is VAPB-PTPIP51. It was through a yeast two-hybrid screen looking for interactors of the ALS-associated vesicle-associated membrane protein B (VAPB) that the interaction between VAPB and protein tyrosine phosphatase-interacting protein 51 (PTPIP51) was first identified (De vos et al., 2012). VAPB is an ER-resident protein whose cytoplasmic domain binds to the cytoplasmic domain of the OMM-resident PTPIP51. Knockdown of either VAPB or PTPIP51 reduces, and overexpression of either protein increases, ER-mitochondrial contacts detectable by EM or confocal microscopy (Stoica et al., 2014; Gómez-Suaga et al., 2019), providing evidence that these proteins form structural tethers between these organelles.

Recently, PDZD8, a paralog of the yeast ERMES protein Mmm1, has been found to be an important player in regulating ER-mitochondrial contacts in mammalian cells. Similar to Mmm1, PDZD8 localizes to ER and ER-mitochondrial contact sites (Hirabayashi et al., 2017). Focus ion beam–scanning EM of control and PDZD8 knockout HeLa cells reveals an ∼80% reduction in ER-mitochondrial contacts, with no detectable effect on the organization of ER or mitochondrial networks. More studies are now required to identify the mitochondrial protein to which PDZD8 binds to mediate ER-mitochondrial contacts.

### Where Are ER-Mitochondrial Contacts Within Neurons?

Whilst ER-mitochondrial contacts have been observed for 60+ years, it is only very recently that MAM structure within neurons has begun to be investigated. Initially, EM analysis of developing and adult hippocampal neurons revealed ER contacts covering ∼10% of the mitochondrial perimeter (Hedskog et al., 2013). Most recently, focused ion beam scanning electron microscopy of mouse brain tissue has revealed that MAMs are abundant throughout neurons, covering 4–5% of the mitochondrial surface (Wu et al., 2017). In fact, ER contacts with mitochondria are the most abundant ER contacts within axons and dendrites where the ER can be seen to wrap around mitochondria.

Known proteins involved in ER-mitochondrial tethering, Mfn2, VAPB-PTPIP51 and PDZD8, are all highly expressed in neurons throughout the brain of developing and adult mice (Eura et al., 2003; Stoica et al., 2014; Hirabayashi et al., 2017). Indeed, MAM-associated proteins have been observed within the ER-mitochondrial interface within synaptic regions, where interactions between VAPB and PTPIP51 have been shown to modulate dendritic spine morphology and synaptic activity in rat hippocampal neurons (Hedskog et al., 2013; Gómez-Suaga et al., 2019). The prevalence of axonal ER-mitochondrial interactions, together with the axonopathies which occur when ER-mitochondrial proteins are mutated, indicates that these contacts mediate functions which are critical to maintain intracellular homeostasis.

### What Are the Functions of ER-Mitochondrial Contacts in Neurons?

Endoplasmic reticulum-mitochondrial contacts are functionally active sites at which several cellular processes occur including: calcium transfer from ER stores to mitochondria; mitochondrial fission; autophagosome formation; lipid biosynthesis; inflammation; apoptosis; ER stress. We will focus here on those functions that have are currently known to be physiologically relevant in neurons.

The most extensively studied function of MAMs within neurons is the regulation of Ca^2+^ signaling which has a fundamental role in the regulation of synaptic transmission and plasticity. Neurons have developed an intricate signaling pathway to couple Ca^2+^ signaling between the ER and mitochondria through specialized domains, permitting mitochondria to take advantage of localized Ca^2+^ release from the ER. Ca^2+^ released from ER stores can be imported into mitochondria via VDAC channels situated on the OMM, however, this requires localized high concentrations of Ca^2+^ (Giacomello et al., 2007). Mitochondria use this Ca^2+^ to generate ATP via the tricarboxylic cycle (Clapham, 2007). ER-mitochondrial contacts are vital for this Ca^2+^ transfer as expression of synthetic linker proteins, which increase the proportion of mitochondria in contact with the ER, increase ER-mitochondrial Ca^2+^ transfer (Csordás et al., 2010). Conversely, loss of PDZD8 or VAPB-PTPIP51 in cultured neurons, which decreases ER-mitochondrial contacts, inhibits ER-mitochondrial Ca^2+^ transfer (Hirabayashi et al., 2017; Gómez-Suaga et al., 2019). This Ca^2+^ shuttling at ER-mitochondrial contacts is vital for the function of neurons. Loss of VAPB-PTPIP51 decreases synaptic activity and number of active spines in cultured hippocampal neurons (Gómez-Suaga et al., 2019). Interestingly, this same study has found that synaptic activity increases ER-mitochondrial contacts pointing to an important role for ER-mitochondrial contacts in regulating synaptic function.

The first direct evidence that mitochondrial fission is regulated at sites where ER and mitochondria interact came from a seminal study by the Voeltz laboratory. Using 3D-EM, ER tubules were observed to wrap around mitochondria constricting the mitochondrial membrane (Friedman et al., 2011). Time-lapse imaging of these constriction sites in mammalian cells show that ER-mitochondrial contacts precede the recruitment of mitochondrial fission factors Drp1 and Mff and that following fission, both daughter mitochondria retain their contacts with the ER (Friedman et al., 2011; Elgass et al., 2015). Due to the exceptionally long nature of axons, mitochondrial processes need to be tightly regulated in order to maintain neuronal health, which need a healthy supply of mitochondria to meet the high energy demands required to maintain synaptic function. Mitochondria in axons are relatively short, ∼1 μm long compared to ∼4 μm long in dendrites, with fission tightly controlled. Impaired mitochondrial fission by knocking down Mff causes reduced neurotransmitter release and disrupts terminal axon branching (Lewis et al., 2018). Indeed, the importance of mitochondrial fission within neurons is highlighted by the fact that loss of Drp1 depletes mitochondria from synapses which causes impaired mitochondrial function and a failure to maintain proper synaptic transmission (Verstreken et al., 2005; Sandoval et al., 2014; Oettinghaus et al., 2016). Disruption of axonal ER by knocking down the ER-shaping proteins Arl6IP1 or Reticulon-like 1 (Rtnl1) also leads to impaired mitochondrial fission and neurodegenerative phenotypes which can be partially restored by increased Drp1 expression (Fowler and O’Sullivan, 2016). Together, these studies point to an important mechanism by which ER-mitochondrial contacts regulate axonal function.

Another function of MAMs that is particularly relevant to axonal physiology is the regulation of autophagy. Autophagy is the degradative pathway responsible for removing dysfunctional intracellular components and damaged organelles by sequestering them in double-membraned vesicles called autophagosomes. In the final step of autophagy, autophagosomes fuse with lysosomes, forming autolysosomes, the contents of which are degraded and their components recycled (He and Klionsky, 2009). The efficient initiation and completion of autophagy is essential for neuronal health which is reflected by the fact that a growing number of neurodegenerative diseases are characterized by autophagic dysfunction (Menzies et al., 2017). The formation of the isolation membrane is the first step in autophagy and the ER-mitochondrial interface has been proposed to serve as a nucleation site during the formation of the isolation membrane. The autophagosomal marker Atg14 localizes to MAMs and the isolation membrane can be observed to form at ER-mitochondrial contact sites upon starvation induced-autophagy (Hamasaki et al., 2013). Altering ER-mitochondrial contacts disrupts autophagy such that increasing ER-mitochondrial contacts by expression of synthetic tethers impairs autophagic flux while knockdown of the ER-mitochondrial tethers VAPB-PTPIP51 activates the autophagy-initiating factor beclin 1 and increases autophagosome formation in a neuroblastoma cell line (Gomez-Suaga et al., 2017; Wu et al., 2018). More research is still required to uncover the mechanisms by which ER-mitochondrial contacts regulate autophagy in neurons.

### Evidence That Links ER-Mitochondrial Contacts to HSP

Several lines of evidence have linked ER-shaping proteins REEP1, Reticulon 2 (RTN2), Atlastin and ARL6IP1, mutations in which cause HSP subtypes SPG31, SPG12, SPG3A and SPG61, respectively, to ER-mitochondrial contacts. Many ER-shaping proteins are found to localize to MAMs and expression of REEP1 or RTN2, but not HSP-causing mutant proteins, increases ER-mitochondrial contacts in split luciferase assays (Lim et al., 2015; Cho et al., 2017). Mutations in animal models of SPG3A and SPG61 reduce mitochondrial fission events indicative of defective ER-mitochondrial contacts (Fowler and O’Sullivan, 2016; Liu et al., 2019). Notably, highly elongated and hyperfused mitochondria and impaired mitochondrial fission have also been reported in fibroblasts of SPG31 patients indicating that that disrupted ER-mitochondrial contacts are a feature of this disease (Goizet et al., 2011; Lavie et al., 2017).

Defects pointing to impaired ER-mitochondrial contacts are also a fairly common feature of non-ER-shaping protein models of HSP. iPSCs from patients with SPG15 and SPG48, caused by mutations in spastizin and AP5Z1, respectively, have altered mitochondrial organization and mitochondrial fission (Denton et al., 2018). Furthermore, mitochondrial defects are evident in lympoblasts from patients with SPG28 and SPG56, caused by mutations in fatty acid metabolism proteins DDHD1 and CYP2U1 (Tesson et al., 2012). Taken together, whilst further studies are required to determine the exact consequences of HSP-causing mutations on MAM organization or function, it is becoming apparent that downstream processes dependant on the ER-mitochondrial axis are affected across many forms of HSP.

## ER-Lipid Droplet Contacts in Neurons

Lipid droplets (LDs) are densely packed neutral lipids, predominantly triacylglycerol (TG) and steryl esters (SE), surrounded by a phospholipid monolayer. LDs are used by many cells as fatty acid reservoirs for use in energy production or membrane biogenesis, but also act as a sink for fatty acids that could otherwise be toxic for cells. The lipids themselves are synthesized within the ER, in which the enzymes required to catalyze the biosynthesis of TG and SE reside. The LDs can either remain in contact with the ER or bud off from it to travel into the cytoplasm (Kassan et al., 2013; Wilfling et al., 2013).

### How Are ER-LD Contacts Regulated?

Several proteins have been identified to play a role in initiating and regulating ER-LD contacts. Here we summarize recent findings on the best studied of these: Seipin, Rab18 and Snx14 (Figure 3B).

Numerous studies have shown that Seipin localizes to ER–LD contacts in yeast, insect, and mammalian cells (Szymanski et al., 2007; Salo et al., 2016; Wang et al., 2016). Recent structural analysis of the *Drosophila* Seipin protein revealed that Seipin forms a ring-shaped complex comprised of 12 Seipin monomers (Sui et al., 2018). This Seipin dodecamer consists of 3 distinct regions: short N- and C-terminal segments which orientate toward the cytoplasm; hydrophobic helices which imbed Seipin into the ER membrane; and a large β-sandwich domain, with similarity to lipid-binding proteins, which projects into the ER lumen. Seipin is required for the proper maturation of LDs and loss of Seipin leads aberrant LD morphology, specifically a general marked reduction in LD size with some very large LDs which are deficient in phospholipids (Szymanski et al., 2007; Fei et al., 2008; Salo et al., 2016; Wang et al., 2016). The current model suggests that Seipin complexes within the ER may localize to areas of high neutral lipid concentration where they can anchor the developing LD to the ER (via the N- and C-terminal segments). It is proposed that the ER luminal β-sandwich domain could function to transfer lipids from the ER into bound LDs, however, the mechanism by which this might occur is not yet known (Sui et al., 2018).

Rab18 was originally described as a lipid-droplet localized protein that induced contacts between ER and LDs (Ozeki et al., 2005). More recently, super-resolution live-cell imaging has revealed that activated Rab18 is normally distributed throughout the ER but upon induction of LD formation Rab18, along with another ER-resident protein DFCP1, localizes to discreet sites on the ER (Li et al., 2019). These discreet sites are likely early LD structures within the ER as treatment with TG synthesis inhibitors blocks formation of these sites. The Rab18-DFCP1 complex interacts with ZW10 and ER-resident SNAREs which maintain the ER-LD contact for these developing LDs (Xu et al., 2018; Li et al., 2019). Rab18 and DFCP1 are required for the formation of ER-LD contacts such that loss of Rab18 or DFCP1 reduces ER-LD contacts while overexpression of either protein increases ER-LD contacts (Xu et al., 2018; Li et al., 2019).

Sorting nexin protein 14 (Snx14) resides within the ER via transmembrane domains and characterization of Snx14 mutants revealed neutral lipid metabolism and LD defects (Bryant et al., 2018). During LD maturation upon treatment with fatty acids, Snx14 specifically localizes to ER-LD contacts (Datta et al., 2019). At these contacts, Snx14 remains embedded in the ER via its N-terminal transmembrane domains and binds to the LD surface via a C-terminal amphipathic helix domain (Datta et al., 2019). Loss of Snx14 reduces ER-LD contacts while over-expression of Snx14 increases ER-LD contacts indicating that Snx14 is acting as a protein tether for these contacts.

Current findings suggest that while there is crosstalk between some ER-LD tethers, others seem to function independently of each other. For example, Seipin regulates the growth of DFCP1-Rab18-labelled developing LDs, though it is not known if this control occurs by a direct interaction between these complexes or by an indirect mechanism (Li et al., 2019). In contrast, Seipin and Snx14 seem to function independently of each other to regulate LD morphology as overexpression of Seipin cannot rescue LD defects in Snx14 cells and vice versa (Datta et al., 2019). It will be important to determine whether distinct ER-LD tethers function in discreet regions of the cytoplasm or in response to different stimuli.

### Where Are ER-LD Contacts Within Neurons?

Endoplasmic reticulum-LD contacts have been visualized extensively in yeast, insect and mammalian cell cultures by both electron and confocal microscopy (Novikoff et al., 1980; Wilfling et al., 2013). More recently, the stability of ER-LD contacts has been measured in real time using sub-diffraction limited confocal microscopy revealing that a large proportion of ER-LD contacts are stable with LD remaining associated with the ER as it moves throughout the cytoplasm of mammalian cell cultures (Salo et al., 2016).

In contrast, ER-LD contacts have been reliably visualized in neurons. In fact, while various studies have reported LDs in *Drosophila* axons (Papadopoulos et al., 2015), rodent cortical neurons (Renvoisé et al., 2016) and primary cultures of cortical neurons (Branchu et al., 2017), many other studies do not see LDs within neurons under normal conditions. While LDs are not readily found within neurons, they are clearly present in the glia adjacent to neurons within central and peripheral neuronal structures. Within *Drosophila*, LDs can be seen within epithelial glia surrounding photoreceptors (Liu et al., 2015) and within cortical glia within the ventral nerve cord (Bailey et al., 2015). Moreover, LDs have been found to accumulate within neurons and glia in response to stressors such as nutrient deprivation, hypoxia, increased reactive oxygen species (ROS), or expression of mutations in proteins involved in lipid production (Inloes et al., 2014; Bailey et al., 2015; Liu et al., 2015).

Given the elusive nature of neuronal LDs, it is unsurprising that ER-LD contacts have not so far been studied in neurons. However, ER-LD protein tethers are present in neurons. Seipin (Wang et al., 2018), Rab18 (Wu et al., 2016) and Snx14 (Huang et al., 2014) are all highly expressed in the mouse brain, localizing within neurites in cultured neurons (Huang et al., 2014; Nian et al., 2019). It is therefore likely that ER-LD contacts can be generated and maintained within neurons and may be dysregulated during neurodegeneration.

### What Are the Functions of ER-LD Contacts in Neurons?

As a result of the paucity of information on LDs and ER-LD contacts within neurons, there are no studies validating their functions within neurons generally or axons specifically. Given the clear overlap between ER-LD organization and neurodegeneration in HSP, it seems crucial that future studies aim to determine the mechanisms by which disrupted ER-LD contacts contribute to pathological dysfunction in motor neurons. A recent paper puts forward several hypotheses as to the potential functions of LDs which may be disrupted during neurodegeneration (Pennetta and Welte, 2018). These include that neuronal LD might act to: prevent neuronal lipotoxicity by sequestering fatty acids and transferring them to glia; traffic proteins and lipids required for synapse assembly along the axon; and sequester mutated or aggregated proteins for degradation. While some evidence exists which could support each of these hypotheses, a significant amount of work is now required to determine whether any of these mechanisms are relevant to neurodegenerative disease.

### Evidence That Links ER-LD Contacts to HSP

Mutations in the ER-LD tether Seipin causes autosomal dominant (AD) HSP subtype SPG17, also known as Silver syndrome, a complicated form of HSP which is characterized by amyotrophy in the hands and sometimes lower limbs (Windpassinger et al., 2004). Expression of disease-causing Seipin mutants *in vitro* and *in vivo* triggers ER stress responses with increased production of ER chaperones (Ito et al., 2008; Yagi et al., 2011) which was proposed to be the mechanism by which motor neurons degenerate in disease. As discussed above, it is now clear that Seipin has a crucial role in LD biogenesis at ER-LD contacts (Salo et al., 2016; Wang et al., 2016). To understand the pathophysiological significance of this in HSP, we need to uncover how HSP-causing mutations affect LD biogenesis *in vivo*.

In addition to Seipin, mutations in several genes encoding enzymes which regulate lipid metabolism cause HSP. DDHD1 and DDHD2 are lipid metabolizing enzymes mutations in which give rise to autosomal recessive (AR) HSP subtypes SPG28 (Bouslam et al., 2005) and SPG54 (Schuurs-Hoeijmakers et al., 2012), respectively. Brain tissue from DDHD1^–/–^ or DDHD2^–/–^ knockout mice has disrupted lipid content (Inloes et al., 2014, 2018). Perturbed lipid processing appears to be an important contributor to neuronal dysfunction with large LD accumulations accompanying marked motor impairments in DDHD2^–/–^ mice (Inloes et al., 2014) and evidence of lipid accumulation in the brains of SPG54 patients (Schuurs-Hoeijmakers et al., 2012). Furthermore, mutations in fatty acid 2-hydroxylase (FA2H) and patatin like phospholipase domain containing 6 (PNPLA6) cause complicated AR-HSP subtypes SPG35 and SPG39, respectively (Rainier et al., 2008; Dick et al., 2010) and mutations in carnitine palmitoyl-transferase (CPT1C) cause pure AD-HSP subtype SPG73 (Rinaldi et al., 2015). Animal models of these disorders frequently display LD defects and motor neuron defects (Song et al., 2013; Rinaldi et al., 2015; Li et al., 2019), highlighting the conserved link between LD organization and neuronal function.

Finally, LD defects are frequently detected in models of HSP which are not directly known to function in lipid metabolism or LD biogenesis. Mutations in the ER-shaping proteins REEP1, Atlastin and Spastin have all been shown to disrupt LD formation *in vivo* (Klemm et al., 2013; Papadopoulos et al., 2015; Renvoisé et al., 2016). This likely occurs largely as a result of the defective tubular ER network organization that loss of these proteins causes though specific roles for these proteins in LD organization cannot be ruled out. To that end, HSP-causing M1 Spastin has recently been shown to disrupt LD content by impaired LD-peroxisome contact formation (Chang et al., 2019). Loss of SPG20 (Spartin) or SPG11 (Spatacsin) causes altered LD regulation in mouse embryonic fibroblasts and primary cortical neurons, possibly by impaired LD turnover (Renvoisé et al., 2012; Branchu et al., 2017). Taken together, it is clear that disruption of lipid metabolism and/or storage at the ER-LD interface is a common feature of HSP.

## ER-Endolysosomal Contacts in Neurons

The endolysosomal system is a dynamic network of intracellular membranous compartments that continuously interconvert and play essential roles in nutrient uptake, metabolic control, macromolecular degradation and signaling (Klumperman and Raposo, 2014). According to their functions, several different types of compartments have been identified, which include early endosomes, late endosomes, recycling endosomes and lysosomes. The maturation of early endosomes into late endosomes is characterized by a series of defined stages, including endosome growth and acidification, which creates a unique environment within the cell to allow for the breakdown of products into smaller polypeptides to help maintain amino acid pools and energy balance. The ER makes dynamic contacts with endosomal compartments which increase as endosomes mature (Friedman et al., 2010, 2013).

### How Are ER-Endolysosomal Contacts Regulated?

Multiple protein tethers regulating contacts between the ER and endosomes/lysosomes have been identified. The best studied of these involve VAPs localized to the ER and FFAT domain-containing proteins ORP1L, StARD3 and StARD3NL localized on endosomes (Loewen et al., 2003). Several other proteins have more recently been shown to contribute to ER-endolysosomal contacts including MOSPD2, protrudin-PI(3)B and PTP1B-EGFR (Figure 3C).

The first ER-endoslysosomal contact site to be characterized in mammalian cells were contacts between the integral ER protein VAPA and the late endosomal cholesterol binding protein ORP1L. ORP1L encodes a member of the oxysterol-binding protein (OSBP) family, a group of intracellular lipid receptors which are characterized by a C-terminal sterol binding and FFAT domains (Loewen et al., 2003). ORP1L is recruited to late endosomes via its interaction with Rab7 where it modulates ER-late endosome interactions. Under conditions of low cholesterol, the cholesterol-binding domain of ORP1L is unoccupied favoring a conformation that promotes interactions between the FFAT domain of ORP1L and VAPA forming ER-late endosomal contacts (Rocha et al., 2009). In addition to ORP1L, two other FFAT domain containing late endosomal proteins StARD3 (steroidogenic acute regulatory domain-3) and StARD3NL (StARD3 N-terminal like) have been shown to modulate ER-late endosomal contacts by directly interacting with VAPA and VAPB proteins (Alpy et al., 2013). Overexpression of ORP1L, StARD3 or StARD3NL increases both the size and number of ER-late endosome interactions, pointing to a crucial role of these proteins in the formation of contacts between these two organelles (Rocha et al., 2009; Alpy et al., 2013). However, whilst the number of contacts increases upon overexpression of these proteins, no studies have reported a reduction in contact site formation upon depletion of ORP1L, StARD3 or StARD3NL. Therefore, whilst is remains unclear whether these proteins function to stabilize or promote ER-endosomal contacts, it is likely that other tethering systems exist to compensate for the absence of VAP proteins. In line with this, a study using a proteomic approach has recently identified MOSPD2 (motile sperm domain containing 2) as a novel ER-anchored protein which binds FFAT motifs and consequently allows membrane tethering *in vitro*, where silencing of MOSPD2 leads to a reduction in the surface contact between ER and endosomes (Di Mattia et al., 2018).

The integral ER protein protrudin, which promotes cellular protrusions and neurite outgrowth in cultured cells (Shirane and Nakayama, 2006; Hashimoto et al., 2014), interacts with PI3P and Rab7 at the endosome to promote ER-endosomal interactions. Overexpression of protrudin causes increased stabilization of tubular SER and the formation of multiple and extended endosomal contacts (Hashimoto et al., 2014; Raiborg et al., 2015). Conversely, reduced protrudin expression disrupts ER organization with an expansion of RER into the periphery of cultured cells (Chang et al., 2013).

Two other proteins reported to promote ER-endolysosomal contacts includes interactions with the endosomal epidermal growth factor receptor (EGFR) and the ER-localized PTP1B (protein tyrosine phosphatase) (Eden et al., 2010). Specifically, the ER has been shown to form direct contact sites with multivesicular bodies (MVBs) where it regulates the trafficking and signaling of the EGFR. These contacts form in both the presence and absence of EGF. Depletion of PTP1B decreases the number of contacts between EGFR-containing MVBs and the ER while overexpression of PTP1B slightly enhanced these contacts suggesting that this protein acts in the formation of the contacts between these two organelles (Eden et al., 2010).

Several additional tethers have been proposed to play a role in regulating contacts between the ER and endosomes, however, it remains unclear whether these serve as functional rather than structural tethers. This includes the endosomal-localized TMCC1 which localizes to discrete sites in the peripheral ER that are spatially and temporally linked to endosomal fission (Hoyer et al., 2018). Additionally, regions in which lysosomes remain closely associated with the ER have been shown to be densely populated by clusters of IP3Rs, however, it is thought that these do not contribute to the assembly of ER-lysosome contacts but rather serve as a functional tether to facilitate Ca^2+^ delivery from the ER to lysosomes (Atakpa et al., 2018).

### Where Are ER-Endolysosomal Contacts Within Neurons?

Contacts between the ER membrane and endosomes, lysosomes and MVBs within yeast and mammalian cells have been visualized by high-resolution electron microscopy (Rocha et al., 2009; Eden et al., 2010; Di Mattia et al., 2018). Within mammalian cells, associations between the ER and endosomes typically cover ∼5% of the endosomal surface, which remain bound to the ER as they traffic (Friedman et al., 2010, 2013). This is achieved through the dynamic nature of ER tubules, which have been shown to rearrange their tubules in order to ensure maintained contact with dynamic endosomes (Friedman et al., 2010). Interestingly, contacts between the ER and endosomes become more tightly associated as they transition through the maturation steps, where endosomes that acquire Rab7, a marker of late endosomes, have been shown to be almost completely bound to the ER membrane (Friedman et al., 2013).

Most known ER-endolysosomal protein tethers have been shown to be expressed in neurons with high levels of expression of VAPA/B (Teuling et al., 2007), FFAT proteins (Johansson, 2003), protrudin (Shirane and Nakayama, 2006) and PTP1B (Song et al., 2016) found in rodent and human brain tissues. Despite this, it is only recently that ER-endolysosomal contacts have been examined within neurons. Focused ion beam scanning electron microscopy of mouse brain tissue reveals extensive contacts between the ER and endosomes, lysosomes and MVBs throughout neurons (Wu et al., 2017). Within neuronal cell bodies the ER was found to contact ∼2% of endosomal and lysosomal membranes while in dendrites and axons the extent of ER-endosomal contacts were somewhat less (1–2%) but were still clearly evident (Wu et al., 2017).

### What Are the Functions of ER-Endolysosomal Contacts in Neurons?

Several different cellular functions have been attributed to ER-endolysosomal contacts including the regulation of endosomal tubule fission and endosome transport as well as lipid and Ca^2+^ transfer between these two organelles. The functions of these contacts have mostly been studied in non-neuronal cells, but recent studies have confirmed some of these functions in neurons.

The endolysosomal system is highly dynamic and ER-endolysosomal contacts play an important role in the regulation of endomembrane fission and vesicle budding. This function was first uncovered in Cos7 cells where contacts between the ER and early and late endosomes were found to mark sites of endosomal constriction and fission (Rowland et al., 2014). Disruption of the SER network by dysregulated expression of the ER-shaping proteins RTN4a or Spastin impairs endosome fission (Rowland et al., 2014; Allison et al., 2017). The mechanism for how ER-endolysosomal contacts regulate endosomal fission is not fully understood but it requires the action of the ESCRT protein IST1 (increased sodium tolerance 1) which forms a helical complex with CHMP1B (charged MVB protein 1B) to modulate endosomal constriction by promoting positive membrane curvature (McCullough et al., 2015; Allison et al., 2017). Additionally, the WASH complex, an actin-regulating complex that is recruited to endosomes by interactions with the retromer complex, has recently been shown to regulate endosomal fission through via interactions with VAP-proteins which act directly at the ER-endosome interface (Derivery et al., 2009; Dong et al., 2016). ER-endolysosomal contacts similarly regulate endolysosomal fission in neurons as disruption of the SER network in mouse primary cortical neurons results in elongated lysosomes, indicative of impaired fission (Allison et al., 2017).

Endoplasmic reticulum-endolysosome contacts are also important in controlling the association of endosomes with the cytoskeleton (Nielsen et al., 1999). Endosomes destined for lysosomal fusion and degradation are trafficked along the cytoskeleton as they undergo many sorting, fusion and fission events. The ER-endosomal tethering complex VAPA-ORP1L modulates the positioning and transport of late-endosomes by forming a complex with Rab7 and its effector RILP (Rab7-interacting lysosomal protein) (Tong et al., 2019). RILP in turn binds the p150^Glued^ subunit of the dynactin protein complex, and under low cholesterol in the endocytic pathway, VAPA-ORP1L interactions at ER-endosome contacts result in VAPA-mediated dissociation of RILP from p150^Glued^ and its associated motors (Rocha et al., 2009; Tong et al., 2019). Additionally, VAPA-ORP1L contacts have been shown to modulate the positioning and transport of autophagosomes by forming ER-autophagosome contacts that prevent transport by the Rab7-RILP-dynein complex (Wijdeven et al., 2016). Given the unique morphology of neurons, the directed traffic of cargo along axons poses a significant challenge which must be tightly regulated in order to maintain neuronal homeostasis. However, to date the role of ER-endolysosome contacts in endosomal trafficking has not been extensively investigated within neurons. The one exception would be protrudin-Rab7-PI(3)B-mediated ER-endolysosome contacts which are required to load the motor protein kinesin-1 on another Rab7 effector FYCO1, allowing for the plus-end transport of late endosomes (Pankiv et al., 2010). Knockdown of protrudin in the neural crest-derived PC12 cell line impairs trafficking of synaptic vesicles from the cell body along neurites providing evidence that ER-endolysosomal contacts do contribute to endosomal trafficking in this neuronal model (Shirane and Nakayama, 2006).

Given that most interactions between the ER and late endosomes involve cholesterol-binding proteins, it is not surprising that an emerging function attributed to ER-late endosomal contacts involves the transfer of sterols between these two organelles. This originally came from the observation of ER-endolysosomal contact sites that were linked with the flow of cholesterol between these two organelles, and most recently, a more directed manner of cholesterol transport has now been shown to occur through these contact sites (Du et al., 2011; Olkkonen and Li, 2013). These include the tethering complexes VAP-ORP1L and VAP-StARD3, where loss of either of these have been shown to impair cholesterol transfer from endosomes to the ER (Wilhelm et al., 2017; Zhao and Ridgway, 2017). However, whilst the functions of these have been extensively studied within non-neuronal cells, whether or how these tethering complexes mediate cholesterol transfer from endosomes to the ER within neurons remain undetermined. Similarly, whilst the bidirectional amplification of ER and lysosome Ca^2+^ signals has been shown to occur at ER-lysosomal contact sites within non-neuronal cells, it remains unclear whether or how ER-lysosomal Ca^2+^ signaling within neurons is mediated. Given that Ca^2+^ signaling between the ER and lysosomes are widely believed to promote endosome–lysosome fusion, endolysosomal trafficking and endosome refilling (Calcraft et al., 2009; Galione et al., 2010; Cao et al., 2017; Feng and Yang, 2017), further studies are required in order to determine whether any of these mechanisms are relevant to maintaining neuronal homeostasis.

### Evidence That Links ER-Endolysosomal Contacts to HSP

Disruption in the organization and/or function of the endolysosomal network is a hallmark of *in vivo* and *in vitro* models of several forms of HSP. Loss spatacsin or spastizin, associated with AR-HSP subtypes SPG11 and SPG15, respectively, results in depletion of the number of lysosomes available for fusion with autophagosomes (Chang et al., 2013; Varga et al., 2015). Loss of the WASH complex protein strumpellin, mutations in which give rise to AD-HSP subtype SPG8, similarly results in a reduction in lysosomal number and a significant enlargement of lysosomes (Allison et al., 2017; Song et al., 2018). Moreover, fibroblasts from SPG48 patients, caused by mutations in the AP-5 subunit AP5Z1, exhibit an accumulation of endolysosomes containing aberrant storage material (Hirst et al., 2015).

There is also mounting evidence for a conserved role for HSP-causing ER-shaping proteins in endolysosomal organization. *In vivo* models of HSP caused by loss of the ER-shaping proteins Spastin, Atlastin and REEP1 give rise to endosomal or lysosomal abnormalities. Time-lapse imaging of tagged endosomes in Arabidopsis *rhd3* mutants, the plant homolog of mammalian Atlastins, reveals that loss of rhd3 significantly disrupts endosomal streaming (Stefano et al., 2015). Primary cortical neurons derived from knockout Spastin or REEP1 mice develop significantly enlarged lysosomes containing membranous material and similar lysosomal abnormalities have been observed in iPSC-derived neurons from a SPG4 patient (Allison et al., 2017). It is interesting to speculate that these phenotypes may arise because of impaired endosomal budding or trafficking but to date there is no direct evidence to support this hypothesis. It is clear however, that impaired autolysosomal clearance results in the accumulation of undegraded material which may be of relevance to disease pathogenesis.

Finally, the ER-endolysosomal protein tether Protrudin has been linked directly to AD-HSP subtype SPG33 (Mannan et al., 2006). It should be noted, however, that the role of Protrudin in HSP is debated as the missense mutation associated with SPG33 has been identified in a SNP in several population and does not lead to loss of function of Protrudin (Martignoni et al., 2008).

## ER-Plasma Membrane Contacts in Neurons

Contacts between the ER and plasma membrane (PM) are ubiquitous in neuronal and non-neuronal cells. These contacts appear to be highly heterogeneous, being made up of various structural and functional proteins and performing a variety of roles depending on both the type and state of the cell.

### How Are ER-PM Contacts Regulated?

Quite a large number of proteins have been shown to localize to ER-PM contacts where they contribute to the maintenance of cellular Ca^2+^ and lipid homeostasis, adequate response to extracellular stimuli or organelle dynamics. Here, we will summarize the proteins known to act as ER-PM structural tethers but not the purely functional, regulator or sorting proteins which also localize to these contacts (Figure 3D).

The integral ER proteins VAPA and VAPB have been mentioned in previous sections functioning as ER-mitochondrial and ER-endolysosomal tethers. The VAPs also mediate ER-PM contacts via interaction with non-conducting Kv2 channels in neurons. Kv2 channels (Kv2.1 and Kv2.2) are voltage-gated potassium channels, abundantly expressed in the mammalian brain, that contain a proximal restriction and clustering (PRC) motif, phosphorylation of which dynamically modulates clustering of Kv2-channels in the PM (Lim et al., 2000). While freely diffusing Kv2 channels regulate neuronal electrical activity, clustered Kv2 channels are non-conducting and seem to act as trafficking hubs (Fox et al., 2013). The PRC motif also functions as a non-canonical FFAT motif by which Kv2 channels bind to VAPs at ER-PM contacts (Johnson et al., 2018; Kirmiz et al., 2018). Overexpression of GFP-tagged Kv2.1 causes a nearly 10-fold increase in the proportion of PM in contact with the ER in HEK cells demonstrating a clear role for Kv2 channel clusters in ER-PM contact formation (Fox et al., 2015).

The extended-synaptotagmins (E-Syts1-3 in mammals) are highly conserved ER-anchored proteins that mediate ER-PM contacts. These contacts are formed by the E-Syt phospholipid-binding C2 domain interacting with the PM phospholipid phosphatidylinositol-4,5-bisphosphate (PI(4,5)P2) (Giordano et al., 2013). E-Syt’s interaction with the PM is regulated by Ca^2+^ such that Ca^2+^ binding to E-Syt releases the C2 domain from an inhibitory binding or conformation so that it can interact with PI(4,5)P2 at the PM (Bian et al., 2018). E-Syts clearly have an important role in ER-PM formation as overexpression of the E-Syts increases, and knockdown decreases, the extent of ER-PM contacts (Giordano et al., 2013). Moreover, the distance between the ER and PM at ER-PM contacts can be regulated by localization of different E-Syts and by [Ca^2+^] (Fernández-Busnadiego et al., 2015).

Transmembrane protein 24 (TMEM24) is an ER-resident protein which forms contacts with the PM. It consists of an N-terminal transmembrane domain, by which it anchors to the ER, and a C-terminal region by which if forms electrostatic interactions with the PM (Lees et al., 2017). Overexpression of TMEM24 increases the proportion of PM in contact with the ER in both HeLa cells and primary neurons (Lees et al., 2017; Sun et al., 2019). This protein tether mediates ER-PM contacts in an activity-dependent manner.

Several proteins are also known to localize to and carry out important functions at ER-PM contacts but have not yet been shown be required to form these contacts. The ER resident stromal interacting molecules STIM1 and STIM2 translocate to the PM upon Ca^2+^ depletion from the ER to bind and activate the PM Orai protein (ORAI1 and ORAI2). STIM-ORAI binding opens the calcium release-activated channels thereby triggering Ca^2+^ entry into the cell, a process known as store-operated Ca^2+^ entry (SOCE) (Park et al., 2009). While Ca^2+^ depletion causes STIM to move toward the PM to interact with ORAI, it is the action of E-Syt1 that seems to mediate the extended ER-PM contacts that occur during Ca^2+^ store replenishment (Poteser et al., 2016; Kang et al., 2019). In addition, several members of the oxysterol-binding protein–related protein (ORP) family localize to ER-PM contacts. Of these, ORP5 an ORP8 possess a transmembrane domain enabling them to directly insert into the ER membrane, while ORP3 and ORP6 lack this transmembrane domain and interact with VAPs within the ER to mediate the ER-PM contacts (Chung et al., 2015; Mochizuki et al., 2018). ORP proteins bind PM phosphatidylinositol lipids through a pleckstrin homology domain and act as lipid transporters, but, no direct evidence exists that these proteins contribute to the structure of ER-PM contacts. Finally, Sec22b is an ER protein which interacts with synataxin 1 (Stx1) at ER-PM contacts without triggering fusion of the two membranes. Artificially elongating Sec22b increases the distance between ER and PM at contacts, but again there is not definitive evidence that Sec22b-Stx1 are required for ER-PM contact formation (Petkovic et al., 2014).

### Where Are ER-PM Contacts Within Neurons?

Endoplasmic reticulum-PM contacts are abundant within neurons having first been reported by electron microscopy studies in the 1960s (Rosenbluth, 1962). This is most clearly evident in the neuronal cell body where ∼12% of the PM surface appears in contact with the ER (Wu et al., 2017). These contacts are not static and are reversibly decreased following neuronal activity (Tao-Cheng, 2018). Within axons and dendrites ER-PM contacts are much less prevalent, perhaps reflecting a much greater PM-to-cytoplasmic ratio in these neuronal compartments (Wu et al., 2017). Nonetheless, ER-PM contacts are present along the length of axons in mammalian neurons.

The Kv2 channel proteins Kv2.1 and Kv2.2 are highly expressed in neuronal tissue and they colocalize with VAP proteins at ER-PM contacts in the cell bodies and axon initial segments of cultured and *in vivo* neurons (Kirmiz et al., 2018; Lebowitz et al., 2019). The interaction between Kv2 channels and VAPs seems to be an important determinant in the localization of these proteins in neurons with overexpression of Kv2.1 causing VAPs to redistribute to large ER-PM clusters while loss of VAPs reduce Kv2 clustering in the PM (Johnson et al., 2018; Kirmiz et al., 2018). Kv2 clusters in motor neurons can also be seen to be dynamic, redistributing into smaller clusters following neuronal activity (Romer et al., 2019).

In non-neuronal cells, E-Syt1 localizes throughout the ER but rapidly translocates to the ER-PM upon increase in cytosolic [Ca^2+^] (Saheki et al., 2016). Within neurons, the E-Syts are expressed in the adult mammalian brain, though only at modest levels (Min et al., 2007; Herdman et al., 2014). However, high levels of E-Syt expression has been reported in the motor neurons of mice and *Drosophila* (Meyer, 2014; Kikuma et al., 2017). The subcellular distribution of E-Syts in neurons has not yet been elucidated.

TMEM24 is highly expressed in neurons and its expression is increased in mature rather than developing neurons (Sun et al., 2019). In both neuronal and non-neuronal cells, TMEM24 localizes throughout the ER but is enriched at ER-PM contacts (Lees et al., 2017; Sun et al., 2019). TMEM24 staining is particularly evident in cell bodies but is faintly detectable in the axons of primary cultured neurons. Synaptic activity triggers the transient removal of TMEM24 from ER-PM contacts and dispersal through the ER (Sun et al., 2019), which corresponds with the observed activity-dependent reduction in the area of ER-PM contacts (Tao-Cheng, 2018).

### What Are the Functions of ER-PM Contacts in Neurons?

Several functions have been ascribed to ER-PM contacts, the best studied of which are their roles in Ca^2+^ regulation, lipid transfer and as trafficking hubs.

The ER acts as a Ca^2+^ sink, providing the internal stores of Ca^2+^ required to propagate Ca^2+^ waves along axons and to stimulate the release of synaptic vesicles from presynaptic terminals. An important role of ER-PM contacts is replenishment of these ER Ca^2+^ stores by SOCE. In *Drosophila*, loss of E-Syt impairs presynaptic neurotransmitter release, despite a comparable number of synaptic vesicles, suggesting that E-Syt-mediated ER-PM contacts function to regulate Ca^2+^-driven synaptic vesicle release (Kikuma et al., 2017). However, a similar function has not been confirmed in mammalian studies. In fact, E-Syt1-3 triple knockout mice do not have any reported brain abnormalities (Sclip et al., 2016), though disruption of SOCE has been linked with several neurodegenerative disorders as reviewed recently (Secondo et al., 2018).

As discussed previously, the ER produces lipids which are then transported throughout the cell either in packaged LDs or by direct transfer which can occur at ER-PM contacts. Furthermore, lipid metabolites are returned from the PM to the ER for recycling. The ORPs, which localize to ER-PM contacts regulate lipid levels by transporting phosphatidylinositol (PI) from the PM to the ER and phosphatidylserine (PS) from the ER to the PM. Altered expression of ORP5 or ORP6 disrupts PI and PS levels at the PM in neurons and non-neuronal cells (Chung et al., 2015; Mochizuki et al., 2018). TMEM24 has also been shown to transport PI from the ER to the PM *in vitro*, though this function has yet to be validated in neurons (Lees et al., 2017). E-Syts may act to link the ER-PM contact functions in Ca^2+^ and lipid transfer as they have been found to transfer various lipids between membranes but only when Ca^2+^ is bound to the C2 of the E-Syt (Yu et al., 2016; Bian et al., 2018). However, this function has also not yet been validated in neurons.

Endoplasmic reticulum-PM contacts mediated by VAP-Kv2.1 interactions form micro-domains within the PM which serve as important trafficking hubs for intracellular signaling. Monitoring sites of single channel insertion into the PM reveals that the majority of endocytosis and exocytosis of ion channels occurs at the perimeter of Kv2.1-labelled ER-PM contacts in cultured neurons (Deutsch et al., 2012). Furthermore, increased expression of Kv2.1, known to drastically increase ER-PM contacts (Fox et al., 2015), decreases the PM mobility and internalization of the dopamine transporter DAT (Lebowitz et al., 2019). Given that Ca^2+^ channels including STIM1-ORAI and CaV1.2 also localize to Kv2.1 clusters, ER-PM contacts may function regulate to activity-dependent trafficking within neurons (Fox et al., 2015).

### Evidence That Links ER-PM Contacts to HSP

To date there is no direct evidence that ER-PM contacts are disrupted in HSP. No known HSP-causing gene product acts as an ER-PM tether, nor have ER-PM contact defects been reported in HSP models. This may reflect the fact that disruption of this contact is not a common feature of disease or maybe that this contact site has not yet been studied in HSP. Several HSP-causing proteins have been found to localize to the PM, in addition to other membrane structures. Both Spartin and NIPA1, mutations in which cause SPG20 and SPG6, respectively, can localize to the PM where they function to regulate the internalization and degradation of receptor proteins (Bakowska et al., 2007; Zhao et al., 2008). Furthermore, Protrudin (linked with SPG33) associates with both ER and PM proteins in neurons, however, the role of these interacts has not been investigated (Hashimoto et al., 2014). Further studies will ascertain whether ER-PM contacts play a role in pathogenicity in HSP.

## ER-Microtubule Contacts in Neurons

Microtubules (MTs), like the ER, form an extensive network that extends the length of neurons from the cell body to the synapse. The ER contacts this MT network via cross-bridging proteins to regulate MT remodeling via MT severing and regrowth (Cui-Wang et al., 2012; Kuo et al., 2019). Furthermore, treatment with the MT-depolymerizing drug nocodazole alters the ratio of ER sheets and tubules indicating that ER-MT contacts also contribute to modulation of ER organization in neurons (Lu et al., 2009; Farías et al., 2019; Schroeder et al., 2019).

### How Are ER-Microtubule Contacts Regulated?

Several integral ER proteins mediate direct (CLIMP-63, p180, REEP1, Sec61β and Spastin) and indirect (STIM1) binding to MTs (Figure 3E).

CLIMP-63 (cytoskeleton-linking membrane protein 63) was the first ER-resident protein to be identified as a MT-binding protein. CLIMP-63 binds MTs via a microtubule binding domain (MTBD) which is regulated by post-translational modification of the C-terminal coiled-coil domains such that phosphorylation leads to release of the MT contacts (Klopfenstein et al., 1998; Vedrenne et al., 2005). Overexpression of CLIMP-63 results in thickening of the ER tubules which align along bundled MTs (Klopfenstein et al., 1998). Several other proteins including syntaxin 5, microtubule-binding protein MAP2 and valosin-containing protein/p97-interacting membrane protein (VIMP) can all interact with CLIMP-63 to modulate ER-MT interactions as reviewed elsewhere (Sandoz and van der Goot, 2015). Packed MT bundles also occur when the ER protein p180 is overexpressed. p180 was originally identified as a ribosome-binding protein but was subsequently identified to contain a MTBD. Knockdown of p180 conversely results in a decrease in MT extensions and collapsed ER (Ogawa-Goto et al., 2007). The ER-shaping proteins REEP1-4, but not REEP5 or REEP6, possess a C-terminal MTBD which is sufficient for MT binding. Increased expression of REEP1 enhances SER alignment with MTs (Park et al., 2010). A very similar phenotype of ER tubules aligning with MTs was recently identified in cells over-expressing the ER protein Sec61β. Sec61β contains a cytoplasmic MTBD, loss of which disrupts ER contacts with MT (Zhu et al., 2018). Together these studies indicate that ER-MT contacts mediated by CLIMP-63, p180, REEP1 or Sec61β contribute to the organization of ER tubules along the MT network.

The M1 isoform of the ER-shaping protein Spastin comprises a RHD, by which it embeds into the outer leaflet of the ER lipid bilayer, in addition to a MTBD and an AAA ATPase domain which catalyzes MT severing. The MTBD of Spastin binds directly to the C-terminal tail of tubulin in an ATP-independent manner (White et al., 2007). Once bound to MTs, Spastin can sever tubulin-tubulin interactions generating internal breaks in the MT network (Evans et al., 2005; Roll-Mecak and Vale, 2008). In addition to its severing activity, Spastin has recently been shown to promote MT regrowth. Spastin stabilizes the new minus-end MTs created by its own severing activity, which serve as templates to support new MT growth and allowing for expansion of the MT network (Kuo et al., 2019).

The Ca^2+^ sensor proteins STIM1 and STIM2 localize to the ER and regulate SOCE. Several years ago, STIM1 was shown to bind to the end-binding (EB) proteins that decorate the dynamic plus-end of MTs (Grigoriev et al., 2008). These STIM1-EB1 interactions are dynamic but can be maintained during MT growth or contraction. STIM1-EB1 contacts stimulate the elongation of ER tubules along the growing MTs and provide a mechanism by which ER-MT contacts couple MT organization and ER-derived calcium signals (Grigoriev et al., 2008; Pavez et al., 2019).

### Where Are ER-Microtubule Contacts Within Neurons?

All ER proteins that mediate ER-MT contacts are highly expressed in neurons, however, many of these proteins have different subcellular distributions in neuronal and non-neuronal cells. In non-neuronal cells, CLIMP-63 (Chang et al., 2013), p180 (Ogawa-Goto et al., 2007) and Sec61β (Shibata et al., 2010) expression is concentrated on perinuclear RER sheets. In neurons, CLIMP-63 expression is similarly predominantly in the RER sheets in the cell body and in elongated tubular structures throughout dendrites mediating the contacts between tubular SER and MTs (Cui-Wang et al., 2012; Farías et al., 2019). p180 localizes to neuronal cell bodies and SER tubules in the proximal regions of axons but not dendrites (Farías et al., 2019). GFP-labeled Sec61β is a commonly used SER marker in neuronal axons in vertebrates and invertebrates, however, this may not reflect endogenous localization as GFP tags are known to disrupt translocon association and localization *in vitro* (Shibata et al., 2010).

The ER-shaping proteins REEP1 (Park et al., 2010) and Spastin (Allison et al., 2017) are expressed throughout the ER network of non-neuronal cells, though M1 Spastin is expressed at low levels and localizes to discrete puncta. In neurons, both REEP1 and M1 Spastin are highly expressed in axonal SER tubules where they colocalize with each other and other ER-shaping proteins including Atlastin (Park et al., 2010).

There are 2 homologs of STIM (STIM1 and STIM2) and 3 homologs of EB (EB1, EB2 and EB3) all of which are highly expressed in neurons. Immunohistochemical analysis reveals that STIM1, STIM2 and EB3 localize to the cell body and along the length of cultured hippocampal neurites (Klejman et al., 2009; Pchitskaya et al., 2017). Furthermore, both STIM1 and STIM2 are required for normal neuronal growth and morphology in primary cultured neurons (Pchitskaya et al., 2017; Pavez et al., 2019).

### What Are the Functions of ER-Microtubule Contacts in Neurons?

Several different lines of evidence have shown that ER-MT contacts play an important role in MT remodeling within neurons. This can occur by either directly by MT severing or indirectly by MT stabilization via post-translational modification of MTs. We will focus on ER-MT contacts in axons.

Spastin functions to sever MTs and it is therefore not a surprise that overexpression of Spastin causes increased MT severing leading to complete MT disassembly. Overexpression of Spastin results in loss of the MT network in cultured neurons (Yu et al., 2008; Riano et al., 2009) and in motor neuron axons *in vivo* (Trotta et al., 2004). Contrary to what might be expected, loss of Spastin actually decreases the MT axonal network *in vitro* and *in vivo* (Sherwood et al., 2004; Roll-Mecak and Vale, 2005; Wood et al., 2006). Recently, mathematical modeling of results from purified proteins identified that in addition to MT severing, Spastin functions to promote MT regrowth (Kuo et al., 2019). While this mechanism would explain *in vivo* loss of Spastin results, it will be important to validate whether Spastin carries out this function in axons. Dynamic instability of MTs can be regulated by post-translational modification of tubulin molecules. Loss of p180 reduces tubulin acetylation, a marker of stable MTs, in axons of cultured neurons resulting in more dynamic MTs (Farías et al., 2019). This confirms previous findings in non-neuronal cells that p180 ER-MT contacts function to stabilize MTs (Ogawa-Goto et al., 2007). Regulation of MT remodeling by ER-MT contacts, whether via severing or altered stability, can have important impacts on neuronal function. Defects in the MT network caused by mutations in Spastin gives rise to impaired axonal trafficking of mitochondria and syanptic vescles as well as causing axonal swellings (Kasher et al., 2009; Denton et al., 2014; Plaud et al., 2018) which are likely pathogenic to neurons.

While many studies have focused on the role of ER-MT contacts on MT remodeling, recent work has pointed to an important role for ER-MT contacts in regulating the distribution and organization of tubular ER in axons. Overexpression of CLIMP-63 or treatment with the MT depolymerizing agent nocodazole drastically increases RER within the cell body and reduces the proportion of axonal SER tubules. Conversely, knockdown of CLIMP-63 increases localization of Sec61β in axons suggestive of increased axonal SER tubules (Farías et al., 2019). This provides strong evidence that ER-MT contacts mediated by CLIMP-63 contribute to regulation of the distribution of ER in axons. Within neuronal axons, ER tubules are transported along stable MTs by MT motor proteins kinesin and dynein. Knockdown of family members of either of these motor protein familes disrupts the anterograde and retrograde transport of ER tubules in the axon. In contrast, knockdown of EB1 or EB3 reduces dedritic SER distribution but does not affect SER transport in axons (Farías et al., 2019). Within neuronal dendrites therefore, STIM-EB interactions regulate SER transport by forming ER-MT contacts at the growing plus ends of dynamic MTs. Together, this study has identified that ER-MT contacts contribute to SER localization within axons which may be vital for establishing neuronal polarity.

### Evidence That Links ER-Microtubule Contacts to HSP

Mutations in the ER-MT contact proteins Spastin and REEP1 cause 2 of the most common forms of AD-HSP, SPG4 and SPG31, respectively. This has led to a lot of work to identify the mechanisms by which disease-causing mutations in these genes cause pathogenicity.

The mutation spectrum of Spastin comprises over 300 pathological mutations which include missense, frameshift, splice site, nonsense and deletions. Many of these mutations cause a reduction in the levels of Spastin protein produced or disrupt Spastin’s ability to bind to or sever MTs (Roll-Mecak and Vale, 2008; Denton et al., 2014). Accordingly, some Spastin mutations appear to mediate disease via a haploinsufficiency mechanism (Roll-Mecak and Vale, 2008; Fassier et al., 2013; Denton et al., 2014), while others function through gain-of-function mechanisms (Solowska et al., 2014; Qiang et al., 2019). *Drosophila*, zebrafish and mouse models of SPG4 display mobility defects, disrupted MT network within motor neurons and axonal swellings (Sherwood et al., 2004; Wood et al., 2006; Qiang et al., 2019). Similarly, patient-derived cells exhibit altered MT stability, trafficking defects and axonal swellings (Denton et al., 2014).

SPG31 is predominantly caused by nonsense mutations in REEP1, resulting in truncated or loss of function proteins. Mice models of HSP generated by knockout of REEP1 develop motor dysfunction and axon length is decreased in primary neurons derived from these mice (Beetz et al., 2013; Renvoisé et al., 2016). It is unclear whether these neurodegenerative phenotypes can be attributed to REEP1s role in ER-MT contacts, however, REEP1 has been shown to modulate the neurotoxicity associated with increased MT stabilization. Neurotoxicity caused by overexpression of the MT-binding protein Tau, which functions to stabilize MTs, can be quantified in the *Drosophila* eye. Overexpressing REEP1 rescues, while knockdown of REEP1 exacerbates, Tau-induced neurotoxicity (Appocher et al., 2014). REEP1 interacts with M1 Spastin in axons mediating ER-MT contacts. Given that HSP-causing mutations in REEP1 fail to interact with M1 Spastin, one hypothesis could be that reduced REEP1 disrupts MT severing, which occurs at ER-MT contacts, and the resulting increase in MT stability contributes the axonal degeneration.

## Conclusion

Within neurons, the ER forms an unbroken network of tubules which extends the entire length of the axon, up to 1 m in the longest axons within the corticospinal tract, making extensive contacts with other organelles, the cytoskeleton and the plasma membrane. The continuous nature of the ER allows it to regulate neuron-wide as well as localized functions. As we have discussed here, many of the functions of neuronal tubular ER occur at ER contacts sites including: ion exchange and homeostasis, lipid synthesis and organelle shaping and organization. However, a significant amount of work is still required to elucidate the exact functions of ER contacts within axons. Furthermore the functional repertoire of ER contacts are likely to expand as novel tethers are identified or known tethers are better characterized within neurons. It has become evident that mutations in ER-shaping proteins, the most common cause of HSP, disrupt ER contacts. Therefore to better understand the pathogenic mechanisms underpinning axonopathy in HSP, it is vital that continued research uncovers the function of the ER and ER contacts in neurons.

## Author Contributions

PF, MG-P, and NO’S wrote and edited the manuscript and prepared the figures. JS commented on and reviewed the manuscript prior to submission. All authors contributed to manuscript revision, read and approved the submitted version.

## Conflict of Interest

The authors declare that the research was conducted in the absence of any commercial or financial relationships that could be construed as a potential conflict of interest.
